# Evidence of CD4^+^ T cell-mediated immune pressure on the Hepatitis C virus genome

**DOI:** 10.1038/s41598-018-25559-6

**Published:** 2018-05-08

**Authors:** Michaela Lucas, Pooja Deshpande, Ian James, Andri Rauch, Katja Pfafferott, Elouise Gaylard, Shahzma Merani, Anne Plauzolles, Andrew Lucas, Wyatt McDonnell, Spyros Kalams, Mark Pilkinton, Cody Chastain, Louise Barnett, Amy Prosser, Simon Mallal, Karen Fitzmaurice, Heidi Drummer, M. Azim Ansari, Vincent Pedergnana, Ellie Barnes, Mina John, Dermot Kelleher, Paul Klenerman, Silvana Gaudieri

**Affiliations:** 1 Present Address: School of Medicine and Pharmacology, Harry Perkins Institute and School of Pathology and Laboratory Medicine, University of Western Australia, Crawley, Western Australia Australia; 2 Department of Immunology, Sir Charles Gairdner Hospital and Pathwest, Crawley, Western Australia Australia; 3 Institute for Immunology and Infectious Diseases, Murdoch University, Murdoch, Western Australia Australia; 4 School of Human Sciences, University of Western Australia, Crawley, Western Australia Australia; 5 Division of Infectious Diseases, University Hospital Bern and University of Bern, Bern, Switzerland; 6 Division of Infectious Disease, Department of Medicine, Vanderbilt University Medical Center, Nashville, Tennessee USA; 7 Department of Pathology, Microbiology and Immunology, Vanderbilt University Medical Center, Nashville, Tennessee USA; 8 Peter Medawar Building for Pathogen Research, University of Oxford, Oxford, UK; 9 Department of Immunology and Department of Microbiology, Monash University, Victoria, Australia; 10 Department of Microbiology and Immunology, University of Melbourne at the Peter Doherty Institute for Infection and Immunity, Victoria, Australia; 11 Wellcome Trust Centre for Human Genetics, Oxford, UK; 12 Department of Clinical Immunology, Royal Perth Hospital and Fiona Stanley Hospital, Perth, Western Australia Australia; 13 Department of Clinical Medicine, Trinity College Dublin, Dublin, Ireland; 14 Present Address: Weatherall Institute of Molecular Medicine, University of Oxford, John Radcliffe Hospital, Oxford, UK; 15 Present Address: Department of Dentistry, Faculty of Medicine and Dentistry, University of Alberta, Alberta, Canada; 16 Present Address: Harry Perkins Institute, University of Western Australia, Crawley, Western Australia Australia; 17 Present Address: Department of Medicine, University of British Columbia, Vancouver, BC Canada

## Abstract

Hepatitis C virus (HCV)-specific T cell responses are critical for immune control of infection. Viral adaptation to these responses, via mutations within regions of the virus targeted by CD8^+^ T cells, is associated with viral persistence. However, identifying viral adaptation to HCV-specific CD4^+^ T cell responses has been difficult although key to understanding anti-HCV immunity. In this context, HCV sequence and host genotype from a single source HCV genotype 1B cohort (n = 63) were analyzed to identify viral changes associated with specific human leucocyte antigen (HLA) class II alleles, as these variable host molecules determine the set of viral peptides presented to CD4^+^ T cells. Eight sites across the HCV genome were associated with HLA class II alleles implicated in infection outcome in this cohort (p ≤ 0.01; Fisher’s exact test). We extended this analysis to chronic HCV infection (n = 351) for the common genotypes 1A and 3A. Variation at 38 sites across the HCV genome were associated with specific HLA class II alleles with no overlap between genotypes, suggestive of genotype-specific T cell targets, which has important implications for vaccine design. Here we show evidence of HCV adaptation to HLA class II-restricted CD4^+^ T cell pressure across the HCV genome in chronic HCV infection without a priori knowledge of CD4^+^ T cell epitopes.

## Introduction

The host’s T cell immune response against rapidly mutable pathogens such as Hepatitis C virus (HCV) has the ability to shape viral genomes during the course of natural infection^1–3^. This form of immune-driven selection pressure results in viral adaptations (genetic variations in the viral genome) that have been shown to be associated with specific host human leucocyte antigen (HLA) alleles as these molecules determine the array (restriction) of viral peptides to be presented to the host’s T cells. These HLA-associated changes in the viral genome commonly lie within or are linked to regions targeted by HCV-specific T cells. Accordingly, viral mutations that abrogate HLA-peptide binding, T cell receptor recognition of this complex or disrupt intracellular epitope processing are positively selected in individuals and can become apparent at a population-level as HLA allele-specific viral polymorphisms^4–7^.

So far, studies on HCV have focused on viral adaptation to antigen-specific CD8^+^ T cell immune responses and were identified as HLA class I-associated viral polymorphisms. These putative viral adaptations were used as leads to identify novel antigen-specific CD8^+^ T cell epitopes^8,9^. These viral adaptations commonly resulted in the loss of antigen recognition (‘classical viral escape’), however alternative mechanisms of viral adaptation were also identified, including examples in which viral adaptation was associated with increased interferon gamma (IFNγ) CD8^+^ T cell responses^10^. The importance of viral adaptation to CD8^+^ T cell pressure in the natural course of HCV infection has been demonstrated in humans and primate models (reviewed in^11^), including in rare single source infection cohorts in which existing viral adaptation in the source virus, and de novo viral adaptations in the recipients, influences infection outcome^1,12,13^.

In the context of CD4^+^ T cells, it is known that the collapse of HCV-specific CD4^+^ T cell responses precedes failure of HCV control^14,15^. However, little is known of the effect of viral adaptation to CD4^+^ T cell pressure (restricted by HLA class II) on HCV infection outcome. While evidence for CD4^+^ T cell immune escape is limited in chronic viral infections in humans, mutations within HLA class II-restricted CD4^+^ T cell epitopes leading to loss of immune control have been observed *in vivo* in the LCMV mouse model^16^. In macaques, it has also been shown that T cell escape through mutations in a CD4^+^ T cell epitope precedes spontaneous viral breakthrough in SIVmac239 viremia in an elite controller^17^. Furthermore, autologous viral variation in CD4^+^ T cell epitopes has been observed in both HIV and HCV infection and in some cases has been shown to correlate with changes in T cell function^18,19^. Data from Zambian subjects infected with subtype C HIV provides evidence that specific HLA class II-associated HIV polymorphisms reflect *in vivo* CD4^+^ T cell pressure^20^, akin to what we described for HLA class I and CD8^+^ T cells in HIV^6^ and HCV^4,5^. The study by Erdmann *et al*. also showed that the novel CD4^+^ T cell targets identified using this approach elicited greater immune responses in HIV controllers than non-controllers. This provides evidence that this type of population-based genetic analysis, which reflects host/viral co-evolution, can successfully identify clinically relevant CD4^+^ T cell epitopes.

Here, we examined the host HLA genotypes and autologous HCV sequences from a cohort of viremic subjects (n = 414) to identify statistically significant HLA class II-restricted viral polymorphisms and show that these amino acid changes occur within CD4^+^ T cell epitopes, thus providing the first evidence for HLA class II-restricted viral adaptation in chronic HCV infection in a single source outbreak and a cross-sectional population cohort.

## Results

### Identification of HLA class II-restricted immune pressure in a single source cohort for HLA class II alleles associated with infection outcome

We initially utilised HLA genotype and autologous HCV sequences from chronic HCV-infected subjects (n = 63) in the Irish single source genotype 1B cohort to identify amino acid changes from the source strain that were significantly associated with specific HLA alleles. The importance of using this cohort is the known strong genetic associations between viral clearance and specific HLA class II alleles^21,22^. There were eight sites in the HCV genome that had a statistically significant HLA class II-associated HCV polymorphism (p ≤ 0.01, Table 1), of which one is part of an extended haplotype containing the HLA class I and II alleles HLA-B*08/-DRB1*03/-DQB1*02 that are in strong linkage disequilibrium (LD) (see methods; Supplementary Table 1). Most of the putative adaptation sites represent viral variation away from the source sequence but in some cases the putative viral adaptation pre-exists in the incoming virus (associations with odds ratio (OR) <1 in Table 1). All eight sites were associated with at least one HLA class II allele that had previously been associated with clearance or chronicity in this single source outbreak (i.e. HLA-DRB1*15, -DQB1*06 with clearance and HLA-DRB1*03, -DQB1*02 with chronicity^21^) as well as the HLA-DQB1*03 allele, which has been associated with beneficial outcome in a genome-wide association study^23^.

**Table 1:** HLA class II-associated HCV variations from a single source genotype 1B outbreak.

Protein	Residue	HLA	Consensus	p-value	OR
NS2	834^#^	DQB1*02	H	0.01	16
	834^#^	DRB1*03	H	<0.001	41
	837	DRB1*03	V	0.006	5
	849	DQB1*06	F	0.009	0.1
	940	DQB1*06	R	0.009	0.1
	1011	DQB1*03	L	0.002	0.1
	1011	DQB1*06	L	<0.001	11
	1011	DRB1*15	L	0.001	8
NS5A	2065	DRB1*03	Y	0.007	19
	2138	DQB1*06	K	0.01	5.7
	2138	DRB1*15	K	0.006	6.5
	2356	DRB1*03	E	0.002	25

Alleles associated with clearance (HLA-DQB1*02 and -DRB1*03) and chronicity (HLA-DQB1*06 and –DRB1*15) in this cohort^21^. Possible HLA-DRB1 and –DQB1 haplotypes indicated with a box. OR = odds ratio. N = 63. ^#^Indicates possible extended haplotype with HLA class I allele (HLA-B*08).

As several of the HLA alleles identified in the study have been associated with clinical outcome in this cohort, we next assessed if the sites in Table 1 were also under selection pressure from other known genetic factors associated with HCV infection outcome. Accordingly, we compared the list of associations with our previous analysis of a subset of individuals from this cohort that identified polymorphic sites in the HCV genome with carriage of the protective genotype for Interferon (IFN)λ3 (CC genotype at rs12979860)^1^. In our previous study, variation at position NS2 849 was associated with the protective CC genotype (p = 0.006), which overlaps with the HLA-DQB1*06 association at the same position (Table 1). However, the effects are in the opposite direction; the protective IFNλ3 CC genotype is associated with variation from the source sequence while HLA-DQB1*06, which has been associated with good outcome in this cohort, is associated with maintenance of the source amino acid at this position. Although numbers are small, among those with the IFNλ3 CC genotype and HLA-DQB1*06 (n = 5) all have the source sequence (p = 0.028).

### Identification of HLA class II-associated HCV polymorphisms that mark putative CD4^+^ T cell adaptation sites for the common genotypes 1A and 3A

This population-based genetic approach was extended to analyse HLA genotype and autologous HCV sequences from a cross-sectional cohort of chronic genotype 1A- (n = 215) and 3A (n = 136)-infected subjects. We identified 38 sites across the HCV genome in which the presence of amino acid variation was significantly associated (p ≤ 0.01) with carriage of a specific HLA class II allele (Table 2). In 32/38 associations the adapted amino acid differed from the consensus amino acid at the respective site and is indicated with an odds ratio greater than one. In six cases the HLA class II association with HCV variation has an odds ratio less than one implying the main circulating viruses are adapted to the specific HLA allele at this site and the adapted amino acid is consensus. Importantly, of the 38 sites listed in Table 2, 10 fell within previously described CD4^+^ T cell epitopes with the correct HLA restriction, where known. Furthermore, an analysis of the HLA class II associations in Table 2 in an alternative chronic HCV infection cohort (Boson cohort; genotype 3A; n = 411^3^); showed significant associations with the appropriate HLA restriction for positions E2 628 (p = 0.04) and NS3 1646 (p < 0.001) and trends for the same direction of association at four additional positions (p < 0.1; E2 499, NS3 1278, NS3 1607, NS5A 2283) for genotype 3A. The trend for the association between position E2 480 and HLA-DRB1*1501 (p = 0.05) was with variation from consensus as opposed to the direction of association for the same site in Table 2 (HLA-DRB1*1501 was associated with maintenance of the consensus amino acid at position E480).

**Table 2:** HLA Class II-associated HCV variations for genotypes 1A and 3A.

GT	Protein	Residue	HLA-DRB1*	Consensus	OR	p-value
1A	E2	524^#^	3:01	A	4.5	0.001
		610	15:01	D	0.25	0.005
		641^^^	3:01	E	0.15	0.005
	NS2	923	11:04	A	29	<0.001
		939	13:02	I	9.4	0.006
		958	10:01	D	28	0.001
		975	13:01	V	4.9	0.007
	NS3	1044^^^	14:01	I	11	0.003
		1115^^^	15:01	Q	10	0.007
		1266^^^	11:01	A	13	0.009
		1397^#^	3:01	K	13	0.002
		1495	3:01	K	4.4	0.005
		1636^^^	13:02	T	13	0.001
	NS4B	1964^^^	4:01	I	4.4	0.005
	NS5A	1980^^^	11:01	I	18	0.007
		1984	3:01	I	16	0.003
		2020	4:04	R	43	0.004
	NS5B	2609^^^	4:01	S	6.9	0.002
		2674^^^	13:01	K	2.3	0.002
		2852	3:01	V	13	0.005
3A	E2	480	15:01	P	0.038	0.001
		499	4:04	P	35	0.001
		554	3:01	T	0.15	0.007
		574	13:01	G	18	0.008
		614	15:01	M	4	0.003
		628	11:01	V	7.9	0.002
	NS2	893	3:01	I	15	0.002
		906	4:04	I	15	0.002
	NS3	1278	1:01	I	11	0.007
		1416^#^	15:01	A	14	0.001
		1607^^^	15:01	T	3.1	0.01
		1646^#^	3:01	M	8.4	0.004
	NS4B	1740	3:01	T	6.9	0.006
	NS5A	2283	1:01	P	12	0.006
		2377	15:01	G	0.1	0.002
	NS5B	2605	13:02	E	0.14	0.006
		2753	13:01	R	6.2	0.003
		2757	13:01	R	6.1	0.008

The association is given as the amino acid residue within the polyprotein and the HLA restriction. Overall N = 215 for genotype 1A and N = 136 for genotype 3A. Breakdown of HCV sequence numbers for each protein is shown in Supplementary Table 4. ^Indicates adaptations that fall within published epitopes for genotype 1 and with the same HLA restriction (http://www.immuneepitope.org). ^#^HLA class I-associated variation at same site likely due to LD between HLA class I and II alleles known to form haplotypes. GT = genotype, OR = odds ratio.

Only 10% of the HLA class II-associated HCV variations (4/38) fell at sites that overlapped with the HLA class I associated HCV variations we identified in our previous study^5^ suggesting the majority of the HLA class II associations are not due to LD between HLA class I and II alleles. None of the HLA class II-associated HCV variations overlap with sites associated with resistance to the new direct acting antivirals (DAAs)^24^. Supplementary Table 2 shows the entire list of HLA class I- and class II-associated HCV variations identified using samples from the same subjects and analysis method.

### Distinct adaptation profiles for HCV genotypes

**Figure 1:**
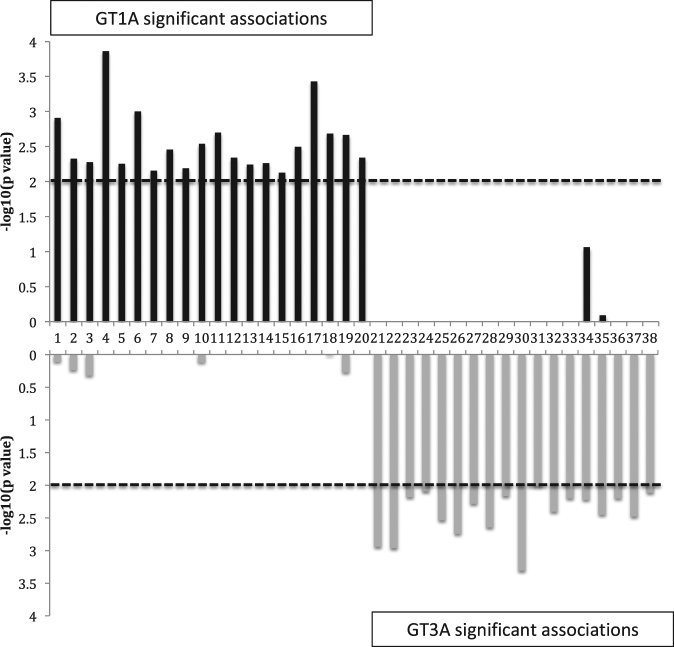
Limited overlap in putative adaptation sites between genotype 1A and 3A. Associations are from Table 2. Note in most cases the alternative genotype is conserved at the site associated with a specific HLA-DRB1 allele in the other genotype and/or the consensus sequence is different. Dashed line represents significance threshold of p ≤ 0.01.

There was no overlap in the position or HLA restriction of the HLA class II-associated HCV variations between the two genotypes, 1A and 3A, and accordingly these putative adaptations may represent genotype-specific T cell targets. Furthermore, for many of these sites the consensus sequence differs between the genotypes and/or there was almost complete conservation in the alternative genotype (29/38). These data concur with our previous report on the limited overlap of the adaptation profiles for the two genotypes based on HLA class I-restricted immune pressure^5^. Figure 1 shows a comparison of the putative adaptation sites for the two genotypes.

### Identification of CD4^+^ T cell epitopes based on HLA class II-associated HCV polymorphisms

**Figure 2:**
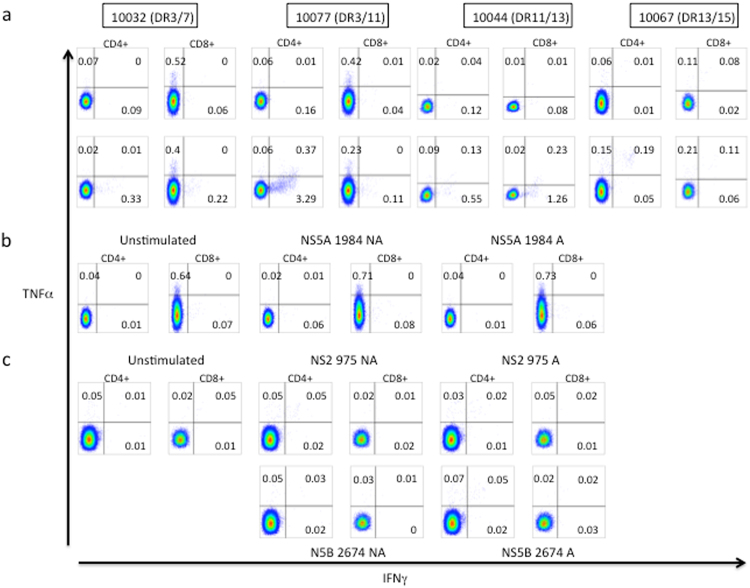
Intracellular cytokine staining indicates HCV-specific CD4^+^ T cell IFNγ response to peptide pool containing putative HLA-class II restricted epitopes. (**a**) TNFα and/or IFNγ production in antigen-specific CD4^+^ and CD8^+^ T cells. Top panel unstimulated and bottom panel peptide pool. (**b**) and (**c**) Specific peptide responses for subject 10032 and 10067, respectively.

As a proof of principle to determine if these putative viral adaptations marked true *in vivo* CD4^+^ T cell epitopes, peptides spanning nine HLA class II (-DRB1)-restricted CD4^+^ T cell genotype 1 epitopes (based on the list of associations in Table 2) were designed using a web-based HLA-peptide binding prediction program (netMHCII; see methods; Supplementary Table 3). Of these targets four had been previously described. Overall, these targets were restricted by six common HLA-DRB1 alleles. To test the peptides we used stored peripheral blood mononuclear cells (PBMCs) from 14 HIV/HCV subjects from a long-term HIV controller cohort based on their HLA alleles (Supplementary Table 5). Peptides relevant to the HLA-DRB1 alleles carried by the HCV-infected subjects were tested in peptide pools. The number of peptides per pool tested ranged from 2–3. Non-adapted and adapted forms of the epitopes were tested in separate pools. Intracellular cytokine staining (ICS) was used to determine cytokine production from antigen-specific CD4^+^ and CD8^+^ T cells. Of the 14 subjects tested, we detected IFNγ responses from CD4^+^ T cells to peptide pools in four subjects (Fig. 2a). Two subjects exhibited positive CD4^+^ T cell IFNγ responses when stimulated with individual peptides representing the putative non-adapted and adapted form of three T cell epitopes from the pool (Fig. 2b,c). Two of the three putative T cell epitopes showed no IFNγ responses when tested with the adapted form of the epitope (Fig. 2b,c). However, for one of the putative T cell epitopes both the adapted and non-adapted form elicited an IFNγ response. Most responses were only positive for IFNγ and did not exhibit production of multiple cytokines (with either TNFα or IL-2; IL-2 data not shown) except for one subject (see Fig. 2c; 10067) in which the CD4^+^ T cell response showed dual TNFα and IFNγ producing CD4^+^ T cells. In two of the four subjects, the peptide pool also elicited IFNγ responses from CD8^+^ T cells. For both of these subjects, the tested peptides included 8–11mers that are predicted to be strong binders to at least one of the HLA class I alleles carried by the subjects (data not shown).

We also tested PBMCs from 14 mono-infected HCV-infected subjects in IFNγ enzyme-linked immunospot (ELISpot) assays. We observed IFNγ responses to three more of the putative epitopes in Supplementary Table 3 but could not confirm them as being elicited from CD4^+^ T cells due to lack of available PBMCs for depletion assays or ICS. As for the ICS results, we also observed IFNγ responses to both non-adapted and adapted forms of the epitopes (see Supplementary material and Supplementary Table 6). However, for these subjects, where CD8^+^ T cell epitopes in the same region(s) were known, we had previously tested the PBMCs with these known CD8^+^ T cell epitopes in these subjects in an earlier study and had not detected responses >25 SFU/million PBMCs^9^.

## Discussion

The outcomes described in this study present evidence for HLA class II-restricted-CD4^+^ T cell induced change across the HCV genome. We show that the putative adaptation sites (identified as HLA class II-associated HCV polymorphisms) likely mark epitopes for CD4^+^ T cells, analogous to what we, and others, have described for HLA class I and CD8^+^ T cell epitopes in HIV^6^ and HCV^4,5,7^. Recent data analyzing HIV adaptation to T cell responses have provided additional evidence to prove that specific HLA class II-associated polymorphisms within the HIV genome exist and are the consequence of *in vivo* CD4^+^ T cell pressure^20^. The data presented here provides first evidence that HLA class II-associated changes within the HCV genome are common and it is likely that these sites are the result of HCV adaptation to *in vivo* CD4^+^ T cell pressure. These results challenge previous reports, including our own, that could not find evidence for viral escape/adaptation (or there was limited variation) in selected CD4^+^ T cell epitopes in longitudinal studies of acute HCV-infected subjects^14,15,25^ and in the primate model^26^. Historically the number of HLA class II epitopes analysed for evidence of CD4^+^ T cell related escape has been small and the possibility that viral adaptation from CD4^+^ T cell responses occurred at different sites cannot be excluded.

Single amino acid variations associated with HLA class II can also lead to the identification of new CD4^+^ T cell epitopes. This approach allows the simultaneous determination of the HLA-restriction of the epitope. So far, the number of CD4^+^ T cell epitopes known for HCV is limited despite the important role of CD4^+^ T cells in anti-HCV immunity^15,27^. As a proof of principle, we synthesized peptides representing epitopes for a number of the putative adaptation sites and were able to confirm three of these epitopes using HLA-matched subjects. It is worth noting that the relatively low HCV-specific responses observed in this study were mainly from chronic infected subjects and similar to outcomes from other groups^15,28^. We have also observed lower responses overall for HCV antigens than for HIV antigens using the same immunological approaches for CD8^+^ T cell responses (median IFNγ response in ELISpot of 590 SFUs/10^6^ cells with an inter-quartile range (IQR) of 280–1440 for HIV versus a median of 63 SFU/10^6^ cells for HCV with an IQR of 38.5–117; including for known T cell epitopes)^8,9^. Other studies have also shown typically lower responses for HCV-specific T cells than for other antigen-specific T cells for HIV, CMV and EBV^15,25,29–34^.

In this study, where possible, peptides containing adapted and non-adapted amino acids were compared. We, and others, have shown for HIV and HCV that amino acid adaptation to HLA class I-restricted CD8^+^ T cell responses can lead to escape as the peptide can no longer be presented by the relevant HLA or recognized by the TCR of the responding effector cell^8,9^. However, we have also shown that in many cases the peptides containing adapted amino acids are still recognized and may even elicit greater IFNγ responses than to the non-adapted form of the peptide^9,10^. These results suggest an alternative mechanism of adaptation that does not result in loss of antigen recognition but rather higher avidity (not tested here) to the adapted form that may act to restrict the host immune response to areas of the virus that can better accommodate variation or may reflect selection of a subset of HCV-specific T cells that are functionally less effective against HCV^10^. Many aspects of viral adaptation to host T cell responses remain undefined but for HIV there is sufficient data to indicate viral adaptation to CD4^+^ and CD8^+^ T cell responses can affect viral control^6,20^.

The immunological consequences of viral adaptation to CD4^+^ T cell escape in HCV infection will be important in the light of the identification of effector CD4^+^ T cells that have direct cytolytic capacity and therefore capable of exerting CD4^+^ T cell-mediated pressure akin to CD8^+^ T cells. Cytolytic CD4^+^ T cells have been shown to be associated with correlates of HIV infection outcome^35^. Interestingly, the importance of the role of CD4^+^ effector T cells in the process of viral clearance may change during infection, as it has been suggested that CD4^+^ T cell-mediated viral lysis is most critical when CD8^+^ T cell responses, T regulatory cells^36^ and B cell responses have failed. This plasticity in the programming of activated CD4^+^ T cell lends itself to strategies to enhance anti-viral immunity. However, the selection of CD4^+^ T cell targets is critical for immunogen design as recently demonstrated by Carlson *et al*.^37^ in a retrospective evaluation of the HIV Step study trial in which the presence of pre-adapted epitopes in the immunogen (to recipients carrying the relevant HLA allele(s)) correlated with deleterious outcomes in HIV infection^37^. In the LCMV model, vaccine-induced CD4^+^ T cell responses alone resulted in immunopathology in vaccinated animals^38^.

As shown for HLA class I-restricted HCV variations^5^, we observe no overlap of association sites for HCV genotype 1A and 3A, highlighting the importance to include genotype-specific T cell epitopes into immunological analyses and future vaccines. No overlap was also observed between the more closely related genotype 1A and 1B putative adaptation sites. However, in this case the genotype 1B sites listed are based on variation from a single source sequence and not a cross-sectional study as performed for genotype 1A (and for 3A) in which the incoming viruses are likely to be different and the distribution of HLA alleles in the chronic cohort are not as affected by the presence of adaptation in a few dominant HLA class I-restricted T cell epitopes. As such, we did not necessarily expect to observe similar adaptation sites for the subtypes in this study but do not exclude overlaps in HCV-specific T cell epitopes. Furthermore, it should be noted that the analysis here is focused on regions of variability to have sufficient power to detect associations and it would be expected that less variable regions would more likely exhibit cross-geno(sub)type targets.

We find no evidence of overlap between HLA class II-associated HCV adaptation sites and sites associated with resistance to the DAAs as was previously shown for HLA class I-associated HCV variations^39^. This suggests that the HCV adaptation sites identified in this study are unlikely to contribute to the presence of resistance mutations in treatment naïve subjects.

In summary, there is evidence for HCV adaptation to CD4^+^ T cell pressure at a population level and our approach may facilitate the more rapid identification and confirmation of T cell targets in HCV. This in turn may aid studies into the biology of T cell failure in untreated HCV infection. Given the analogy of our data to what has been described for HCV adaptation to CD8^+^ T cell pressure, our findings may also indicate that the HCV genome in response to CD4^+^ T cell pressure selects for viral mutations that lead to the evasion of CD4^+^ dependent immune recognition and this may contribute to the failure of CD4^+^ T cells in those with persistent infection.

## Subjects and Methods

### Ethics statement

Written informed consent was obtained from participants. Ethics approval for the conduct of this study was obtained from the Royal Perth Hospital Human Ethics Committee (EC2004/005), Murdoch University Human Research Committee (2014/048) and Vanderbilt University Medical Center (IRB #100061 and #030005). The protocol and the procedures of the study were conducted in conformity with the ethical guidelines of the World Medical Association Declaration of Helsinki.

### Subjects: Chronic HCV-infected subjects for genetic study

Plasma samples were obtained from 63 subjects with chronic HCV infection from a cohort of women who had been infected with an HCV genotype 1B viral strain through the administration of anti-D immunoglobulin as previously described^1,22^. PBMCs, plasma and/or DNA samples were also obtained from individuals with chronic HCV genotype 1A (n = 187) or 3A (n = 136) infection recruited from Australia (n = 145), Switzerland (n = 99; HIV/HCV co-infected), and the UK (n = 79) as previously described^5^. Twenty-eight additional samples were obtained from HIV/HCV co-infected individuals from the Vanderbilt University Medical Center in Tennessee, USA (via the Tennessee Center for AIDS research; CFAR). These subjects were all on anti-retroviral therapy and had undetectable HIV viral load.

### DNA and Viral RNA Extraction

DNA was obtained from whole blood using the QIAamp DNA Blood Mini Kit following the manufacturer’s guidelines. Viral RNA was extracted from plasma samples using either the QIAamp Viral RNA Mini Kit (QIAGEN) or the COBAS AMPLICOR HCV Specimen Preparation Kit version 2.0 (Roche) according to the manufacturer’s instructions.

### HLA genotyping

Two-digit resolution HLA class II typing (HLA-DRB1 and HLA-DQB1) for the single source outbreak cohort was performed at St. James Hospital (Dublin, Ireland)^21^. High-resolution four-digit HLA-DRB1 typing for the chronic HCV-infected genotype 1A and 3A subjects was performed using sequence-based methods as previously described^40^.

### Sanger-based population viral sequencing

Sanger-based bulk sequencing of the HCV genome (E2-NS5B) was performed as previously described^1,5^. Mixtures were identified where the secondary peak was ≥20% of the major peak. Due to the variability of the HCV genome, some samples failed to produce a PCR product and as a result, some individuals did not contribute sequences for all proteins. Supplementary Table 4 contains the breakdown of the number of subjects used per protein in the analyses described below.

### Genotyping of IFNλ3 rs12979860 for the Single source outbreak cohort

Genotyping data of the IFNλ3 rs12979860 SNP was available on 34 subjects of the Irish single source outbreak as described in^1^.

### Statistical Methods

#### HLA Association with Viral Polymorphism

Associations between HLA alleles and amino acid distribution at each residue of the HCV proteins were assessed via Fisher’s exact tests for classification as consensus versus non-consensus amino acid using TIBCO Spotfire S + 8.2 (Palo Alto, CA). Here, consensus refers to the most common amino acid at a single amino acid site.

#### Stratified Analysis by Way of Mantel-Haenszel Tests to assess Phylogenetic Relatedness

In this study, we addressed the issue of a founder effect (where an HLA allele is overrepresented in a subgroup of individuals that have viral sequences sharing a recent common ancestor) by identifying clusters of possibly related sequence and assessing the potential impact of such relatedness by performing analyses stratified by clusters as previously described^5^. Associations between polymorphisms at each amino acid residue and the HLA alleles in the population adjusted for cluster strata were then assessed by Mantel-Haenszel tests. We restricted the analysis to those sites with at least five non-consensus amino acids and there were at least five carriers of the HLA allele. Associations with *p* ≤ 0.01 for both the Fisher’s exact test and the Mantel-Haenszel method were reported. As not all subjects had complete coverage of each protein, a breakdown of those sequences covering more than 90% of the protein is indicated in Supplementary Table 4. For the single source outbreak cohort no correction was applied for founder effects as previously described^1^ and only associations with *p* ≤ 0.01 were reported. Note here we used an adjusted p value of ≤0.01 as a cut-off and HLA-associated HCV variations were not excluded due to q scores (based on a false discovery rate analysis as previously performed^5^ and values shown in Supplementary Tables 1 and 2) as values >0.2 were obtained for known and confirmed HLA class I^5^ and II-restricted HCV T cell epitopes. Furthermore, the cross-sectional nature of part of this analysis (compared to a common infecting source with a more restricted mutational space) and promiscuity of HLA class II binding would likely reduce the ability to detect a signal in these cohorts.

#### Identification of associations that may reflect extended haplotypes

The HLA genes reside within the Major Histocompatibility Complex (MHC); a region known to exhibit extensive LD giving rise to extended haplotypes (ancestral haplotypes) containing specific HLA class I and II alleles^41^. In some cases, HLA class II associations with HCV polymorphism fell at the same site as an association we had previously obtained from our earlier study identifying HLA class I-associated HCV polymorphisms^5^. If these overlaps reflected a likely ancestral haplotype they were indicated as such in the appropriate table.

#### Association analysis using an alternative cohort

To examine the HLA class II associations identified using the approach above in an alternative chronic HCV-infection cohort, a separate analysis was performed on the Boson cohort as described in^3^. This cohort comprised chronic HCV-infected subjects with pre-treatment viral sequence and host genotype of >800,000 SNPs using the Affymetrix UK Biobank array. The four digit host HLA alleles were imputed as reported previously^3^. To limit the impact of population structure we limited the cohort to subjects with self-reported White ancestry infected with genotype 3A virus (n = 411). Principle component analysis was performed on the host SNP data and on the virus nucleotide sequence data and the first two viral PCs and the first 3 host PCs were used as covariates to account for possible hidden population structure. The association testing was performed using logistic regression where the response variable was the presence or absence of the indicated amino acid and the explanatory variable was the presence or absence of the HLA allele and the host and viral PCs as covariates. All the analysis was performed in R (version 3.2.2). This analysis was performed on only a select number of positions within the HCV genome and for specific HLA alleles. No correction for multiple testing was performed and p < 0.05 was considered significant.

#### IFNλ3 associations with HCV polymorphisms

We had previously assessed associations between the presence or absence of the minor allele of rs12979860 of *IFN*λ3 and consensus or nonconsensus amino acids at each residue of the HCV proteins via Fisher’s exact test. Because of the smaller number of subjects with typing available for this part of the analysis, no assessment of false discovery rates was made, and P < 0.01 was used to indicate significance.

### Prediction of CD4^+^ T cell HCV epitopes: selection of peptides

The web-based HLA binding program netMHCII^42^ was used to predict HCV-specific HLA class II-restricted T-cell epitopes based on the list of HLA class II-associated HCV polymorphisms obtained for the genotype 1A and 3A component of the study (Supplementary Table 3). Initially, stretches of 20 amino acids either side of the site of HLA-association was used in the web-based HLA-binding prediction program and hits reflecting weak or strong binding were used to obtain 15–20mer peptides. In order to capture immune responses towards the circulating viral strains within the cohort, consensus and up to three variant versions of peptides were synthesised (Mimotopes or GenScript) based on sequence data from the genetic study^5^.

### Subjects: HCV-exposed subjects for immunology study

PBMC samples from 14 chronic HIV/HCV co-infected subjects in a HIV long-term controller cohort from the Vanderbilt Comprehensive Care Center in Tennessee^43^ were assessed for IFNγ responses to peptides representing putative CD4^+^ T cell epitopes using ICS. These subjects were all chronically HCV-infected (genotype 1) with median CD4^+^ T cell counts of 495 cells/μl (IQR 302–594 cells/μl) and median HIV viral load of 4347 copies/ml (IQR 91–10867 copies/ml) (Supplementary Table 5). These subjects do not overlap with the 28 subjects from the Vanderbilt University Medical Center described above.

### PBMC separation

PBMCs were isolated using the Accuspin System-Histopaque method (Sigma) according to the manufacturer’s guidelines.

### Intracellular cytokine staining (ICS)

For subjects with carriage of the relevant HLA-DRB1 alleles and available PBMCs, ICS with peptide pools was used to examine the phenotype of HCV-specific T cells and cytokine production. These peptide pools contained specific HLA-DRB1 allele-restricted epitopes with a maximum of six peptides per pool at a final concentration of 10μg/ml. The peptide pools tested were dependent on the HLA-DRB1 genotype of the subject. Briefly, approximately 1 × 10x^6^ PBMCs cells were stimulated with peptide pools in the presence of co-stimulatory molecules CD28 and CD49d (BD Biosciences) for two hours before the addition of brefeldin A (GolgiPlug; BD Biosciences) and incubated for a further 12 hours. Cell surface markers and intracellular cytokine production were analyzed using the following monoclonal antibodies: anti-CD3-A700 (BD Biosciences); anti-CD4-PcPCy5.5; anti-CD8-PECF594; anti-CD14-V500; anti-CD19-V500; anti-IFNγ-fluorescein isothiocyanate (FITC); anti-TNFα PE-Cy7; anti-IL2-BV451 pacific blue and anti-PD-1-phycoerythrin (PE) (BioLegend). AquaViD (Life Technologies) was used for the exclusion of dead cells. All samples were acquired using a LSR II flow cytometer (BD Biosciences) and analyzed with BD FACSDiva software (BD Bioscience) or FlowJo v10.2 (FLOWJO). A response was deemed positive if at least three times the background.

## Electronic supplementary material

Supplementary material
