# Supplementary material for: Evidence of CD4+ T cell-mediated immune pressure on the Hepatitis C virus genome
Source: Sci Rep. 2018 May 8;8:7224. doi: 10.1038/s41598-018-25559-6 (PMC5940905; doi:10.1038/s41598-018-25559-6)
Supplement: Supplementary file 1 — Supplementary material [file 41598_2018_25559_MOESM1_ESM.pdf]

**Evidence of $CD4^+$ T cell-mediated immune pressure on the Hepatitis C virus genome**

Michaela Lucas[1,2,3], Pooja Deshpande[4], Ian James[3], Andri Rauch[5], Katja Pfafferott[3,#], Elouise Gaylard[3], Shahzma Merani[4,#], Anne Plauzolles[4], Andrew Lucas[3,#], Wyatt McDonnell[6], Spyros Kalams[6], Mark Pilkinton[6], Cody Chastain[6], Louise Barnett[6], Amy Prosser[3,#], Simon Mallal[3,6,7], Karen Fitzmaurice[8], Heidi Drummer[9,10], M. Azim Ansari[8], Vincent Pedergnana[11], Ellie Barnes[8], Mina John[2,12], Dermot Kelleher[13,#], Paul Klenerman[8], Silvana Gaudieri[3,4,6*]

[1]School of Medicine and Pharmacology, Harry Perkins Institute and School of Pathology and Laboratory Medicine, University of Western Australia, Western Australia, Australia

[2]Department of Immunology, Sir Charles Gairdner Hospital and Pathwest, Western Australia, Australia

[3]Institute for Immunology and Infectious Diseases, Murdoch University, Western Australia, Australia

[4]School of Human Sciences, University of Western Australia, Western Australia, Australia

[5]Division of Infectious Diseases, University Hospital Bern and University of Bern, Bern, Switzerland

[6]Division of Infectious Disease and [7]Department of Pathology, Microbiology and Immunology, Vanderbilt University Medical Center, Nashville, Tennessee, USA

[8]Peter Medawar Building for Pathogen Research, University of Oxford, Oxford, UK

[9]Department of Immunology and Department of Microbiology, Monash University, Victoria, Australia

[10]Department of Microbiology and Immunology, University of Melbourne at the Peter Doherty Institute for Infection and Immunity, Victoria, Australia

[11]Wellcome Trust Centre for Human Genetics, Oxford, UK

[12]Department of Clinical Immunology, Royal Perth Hospital and Fiona Stanley Hospital, Western Australia, Australia

[13]Department of Clinical Medicine, Trinity College Dublin, Ireland

#Current addresses: KP: Weatherall Institute of Molecular Medicine, University of Oxford, John Radcliffe Hospital, Oxford, United Kingdom; SM: Department of Dentistry, Faculty of Medicine and Dentistry, University of Alberta, Alberta, Canada; AL: Harry Perkins Institute, University of Western Australia, Western Australia, Australia; AP: Affiliations[1, 2, 4]; DK: Department of Medicine, University of British Columbia, Vancouver, BC, Canada.

**Supplementary Table 1: HLA class I- and II-associated HCV variations for the single source outbreak cohort.**

| Protein | Residue | HLA | Consensus | p-value | OR | q-value |
|---|---|---|---|---|---|---|
| E2 | 394 | C*06 | A | 0.008 | 11 | 0.74 |
| | 398 | A*01 | S | 0.01 | 0.05 | 0.74 |
| | 401 | C*07 | T | 0.005 | 0.08 | 0.73 |
| | 404 | A*02 | F | 0.004 | 30 | 0.73 |
| | 405 | C*16 | S | 0.006 | 0.04 | 0.73 |
| | 579 | A*01 | D | 0.01 | 0.12 | 0.74 |
| | 627 | B*08 | T | 0.006 | 11 | 0.73 |
| NS2 | 834 | B*08 | H | 0.001 | 15 | 0.04 |
| | 834 | DQB1*02 | H | 0.01 | 16 | 0.26 |
| | 834 | DRB1*03 | H | 0.001 | 41 | 0.02 |
| | 837 | DRB1*03 | V | 0.006 | 5 | 0.18 |
| | 849 | DQB1*06 | F | 0.009 | 0.1 | 0.26 |
| | 940 | DQB1*06 | R | 0.009 | 0.1 | 0.26 |
| | 951 | A*02 | D | 0.003 | 12 | 0.11 |
| | 951 | C*05 | D | 0.004 | 11 | 0.15 |
| | 1011 | DQB1*03 | L | 0.002 | 0.1 | 0.06 |
| | 1011 | DQB1*06 | L | 0.001 | 11 | 0.03 |
| | 1011 | DRB1*15 | L | 0.001 | 8 | 0.06 |
| NS3 | 1040 | B*07 | L | 0.007 | 12 | 0.16 |
| | 1087 | A*03 | T | 0.001 | 17 | 0.03 |
| | 1088 | A*03 | K | 0.001 | 57 | 0.02 |
| | 1130 | B*14 | L | 0.009 | 17 | 0.25 |
| | 1130 | C*08 | L | 0.001 | 36 | 0.03 |
| | 1282 | A*02 | V | 0.005 | 25 | 0.13 |
| | 1282 | B*14 | V | 0.001 | 35 | 0.03 |
| | 1282 | C*08 | V | 0.002 | 26 | 0.06 |
| | 1370 | B*07 | I | 0.002 | 0.1 | 0.07 |
| NS4B | 1958 | C*08 | K | 0.009 | 10 | 0.06 |
| NS5A | 2065 | DRB1*03 | Y | 0.007 | 19 | 0.55 |
| | 2138 | DQB1*06 | K | 0.01 | 5.7 | 0.55 |
| | 2138 | DRB1*15 | K | 0.006 | 6.5 | 0.55 |
| | 2143 | C*16 | E | 0.008 | 17 | 0.55 |
| | 2356 | DRB1*03 | E | 0.002 | 25 | 0.39 |
| NS5B | 2518 | A*03 | K | 0.001 | 11 | 0.03 |
| | 2518 | C*08 | K | 0.005 | 9.7 | 0.20 |
| | 2609 | B*35 | S | 0.001 | 29 | 0.02 |

| | | | | | | |
|---|---|---|---|---|---|---|
| | **2609** | **C*04** | **S** | **0.001** | **19** | **0.02** |
| | **2821** | **B*18** | **R** | **0.005** | **21** | **0.20** |

*Alleles associated with clearance indicated in dark grey and alleles associated with chronicity indicated by light grey shade. Known HLA haplotypes that include class I and II alleles indicated with a box. Note only two-digit resolution HLA typing performed on this cohort.*

**Supplementary Table 2: HLA class I- and II-associated HCV variations for chronic HCV-infected genotype 1A and 3A subjects.**

| GT | Protein | Residue | HLA | Consensus | p-value | q-value | OR |
|---|---|---|---|---|---|---|---|
| | | 824 | C*15:02 | V | <0.001 | 0.34 | 8.8 |
| | | 841 | C*04:01 | W | 0.003 | 0.34 | 19 |
| | | 851 | C*15:02 | T | <0.001 | 0.34 | 24 |
| | | 861 | C*06:02 | V | 0.002 | 0.72 | 9.4 |
| | | 881 | C*12:03 | H | 0.004 | 0.60 | 7.3 |
| | | 923 | DRB1*11:04 | A | <0.001 | 0.47 | 19 |
| | | 939 | DRB1*13:02 | I | 0.006 | 0.82 | 9.4 |
| | | 957 | B*13:02 | R | 0.001 | 0.70 | 6.6 |
| | | 957 | C*06:02 | R | 0.001 | 0.21 | 6 |
| | NS2 | 958 | B*37:01 | D | <0.001 | <0.01 | 880 |
| | | 958 | C*06:02 | D | <0.001 | <0.01 | 33 |
| | | 958 | DRB1*10:01 | D | <0.001 | 0.34 | 30 |
| | | 962 | B*35:03 | N | 0.002 | 0.53 | 19 |
| | | 963 | C*07:01 | G | 0.005 | 0.47 | 3.3 |
| | | 965 | C*07:01 | R | 0.007 | 0.38 | 3.1 |
| | | 975 | DRB1*13:01 | V | 0.008 | 0.80 | 4.9 |
| | | 998 | B*1302 | N | 0.001 | 0.34 | 8.3 |
| | | 1006 | B*3701 | R | <0.001 | <0.01 | 46 |
| | | 1017 | B*1501 | G | 0.003 | 0.34 | 6.7 |
| | | 1044 | DRB1*14:01 | I | 0.004 | 0.74 | 11 |
| | | 1081 | C*05:01 | V | 0.003 | 0.58 | 12 |
| | | 1094 | C*16:01 | K | <0.001 | 0.36 | 9.1 |
| | | 1113 | A*23:01 | A | 0.001 | 0.34 | 27 |
| | | 1115 | DRB1*15:01 | Q | 0.006 | 0.55 | 7.5 |
| | | 1266 | DRB1*11:01 | A | 0.003 | 0.70 | 8.3 |
| 1A | | 1272 | A*32:01 | H | <0.001 | 0.20 | 25 |
| | | 1366 | C*15:02 | A | 0.001 | 0.70 | 12 |
| | | 1368 | B*51:01 | S | 0.003 | 0.70 | 14 |
| | | 1397 | B*08:01 | K | 0.004 | 0.14 | 11 |
| | NS3 | 1397 | DRB1*03:01 | K | <0.001 | 0.05 | 13 |
| | | 1398 | B*08:01 | K | 0.002 | 0.34 | 7.3 |
| | | 1403 | B*08:01 | L | 0.001 | 0.34 | 16 |
| | | 1403 | C*07:01 | L | 0.003 | 0.70 | 10 |
| | | 1444 | A*01:01 | F | <0.001 | <0.01 | 0.13 |
| | | 1444 | B*08:01 | F | 0.003 | 0.57 | 0.24 |
| | | 1495 | A*01:01 | K | 0.003 | 0.18 | 6 |
| | | 1495 | DRB1*03:01 | K | 0.005 | 0.70 | 4.4 |
| | | 1503 | C*12:03 | A | 0.001 | 0.34 | 16 |
| | | 1556 | A*29:02 | E | 0.008 | 0.34 | 16 |
| | | 1636 | DRB1*13:02 | T | 0.007 | 0.74 | 7.6 |
| | NS4A | 1695 | B*27:05 | I | 0.006 | 0.51 | 11 |
| | | 1723 | B*37:01 | M | <0.001 | 0.13 | 38 |
| | | 1747 | C*04:01 | A | 0.006 | 0.51 | 0.17 |
| | NS4B | 1876 | B*40:01 | T | <0.001 | 0.22 | 15 |
| | | 1942 | B*14:02 | A | 0.002 | 0.51 | 18 |

| | | | | | | | |
|---|---|---|---|---|---|---|---|
| | | 1942 | C*08:02 | A | 0.009 | 0.72 | 12 |
| | | 1964 | DRB1*04:01 | I | 0.006 | 0.34 | 4.4 |
| | NS5A | 1980 | DRB1*11:01 | I | 0.009 | 0.63 | 18 |
| | | 1984 | DRB1*03:01 | I | 0.001 | 0.98 | 4.3 |
| | | 2000 | C*04:01 | M | 0.001 | 0.34 | 11 |
| | | 2020 | DRB1*04:04 | R | <0.001 | 0.23 | 16 |
| | | 2024 | C*14:02 | I | 0.007 | 0.34 | 29 |
| | | 2143 | B*18:01 | D | 0.002 | 0.70 | 5.9 |
| | | 2155 | B*35:01 | P | <0.001 | 0.10 | 34 |
| | | 2276 | B*40:01 | R | 0.001 | 0.22 | 24 |
| | NS5B | 2467 | B*15:01 | Q | <0.001 | <0.01 | 9.5 |
| | | 2510 | A*31:01 | S | <0.001 | <0.01 | 94 |
| | | 2530 | B*18:01 | C | <0.001 | 0.34 | 25 |
| | | 2533 | B*27:05 | R | 0.001 | 0.36 | 24 |
| | | 2597 | C*06:02 | D | 0.003 | 0.34 | 19 |
| | | 2609 | DRB1*04:01 | S | 0.002 | 0.70 | 6.9 |
| | | 2629 | B*57:01 | K | 0.002 | 0.70 | 14 |
| | | 2674 | DRB1*13:01 | K | 0.004 | 0.98 | 2.3 |
| | | 2841 | B*27:05 | A | <0.001 | 0.18 | 12 |
| | | 2846 | B*27:05 | M | 0.001 | 0.19 | 12 |
| | | 2852 | DRB1*03:01 | V | 0.006 | 0.59 | 13 |
| 3A | NS2 | 893 | DRB1*03:01 | I | 0.005 | 0.71 | 12 |
| | | 906 | DRB1*04:04 | I | 0.002 | 0.71 | 15 |
| | | 981 | B*44:03 | I | 0.003 | 0.71 | 10 |
| | NS3 | 1133 | A*03:01 | V | 0.007 | 0.71 | 12 |
| | | 1278 | DRB1*01:01 | I | 0.007 | 0.71 | 11 |
| | | 1290 | C*03:04 | K | 0.004 | 0.62 | 11 |
| | | 1409 | B*44:02 | G | 0.009 | 0.77 | 6.7 |
| | | 1416 | B*07:02 | A | 0.006 | 0.65 | 8.5 |
| | | 1416 | DRB1*15:01 | A | 0.001 | 0.37 | 17 |
| | | 1607 | DRB1*15:01 | T | 0.001 | 0.78 | 3.1 |
| | | 1637 | B*44:03 | L | <0.001 | <0.01 | 22 |
| | | 1646 | A*01:01 | M | 0.001 | 0.62 | 8.9 |
| | | 1646 | B*08:01 | M | 0.001 | 0.62 | 11 |
| | | 1646 | DRB1*03:01 | M | 0.004 | 0.71 | 8.4 |
| | NS4B | 1740 | DRB1*03:01 | T | 0.006 | 0.77 | 6.9 |
| | | 1759 | B*57:01 | A | 0.004 | 0.51 | 11 |
| | NS5A | 1982 | B*57:01 | D | 0.003 | 0.71 | 9.1 |
| | | 2143 | A*24:02 | D | 0.006 | 0.71 | 11 |
| | | 2248 | B*35:01 | T | 0.005 | 0.65 | 14 |
| | | 2283 | DRB1*01:01 | P | 0.007 | 0.93 | 12 |
| | | 2320 | A*02:01 | G | 0.004 | 0.75 | 0.24 |
| | | 2377 | A*02:01 | G | 0.006 | 0.37 | 0.19 |
| | | 2377 | DRB1*15:01 | G | 0.004 | 0.62 | 0.18 |
| | NS5B | 2467 | B*15:01 | Q | <0.001 | 0.51 | 17 |
| | | 2467 | C*03:03 | Q | 0.001 | 0.71 | 17 |
| | | 2491 | A*11:01 | V | 0.001 | 0.62 | 17 |
| | | 2605 | DRB1*13:02 | E | 0.001 | 0.94 | 0.14 |
| | | 2753 | DRB1*13:01 | R | 0.003 | 0.71 | 6.2 |
| | | 2757 | DRB1*13:01 | R | 0.001 | 0.93 | 6.1 |

| | | | | | | | |
|---|---|---|---|---|---|---|---|
| | | **2799** | **B*51:01** | **K** | **0.001** | **0.77** | **5.6** |

*Known haplotypes indicated with a box. HLA class II associations in grey. Note high resolution HLA typing on this cohort resulted in four-digit typing for HLA.*

**Supplementary Table 3: Peptides used in ICS assays.**

| ID | HLA Restriction | Sequence |
|---|---|---|
| NS2_923 | DRB1*1104 | LLRICa/tLARKMAGGHYVQMA |
| NS2_975 | DRB1*1301 | HNGLRDLAVAVEPVv/iFSQME |
| NS3_1266 | DRB1*1101 | TLGFGa/vYMSKAHGIDPNIRT |
| NS4B_1964 | DRB1*0401 | LHQWi/vSSECTTPCSG |
| NS5A_1984 | DRB1*0301 | RDIWDWi/vCEVLSDFKTWLKA |
| NS5A_2020 | DRB1*0404 | YRGVWr/qGDGIMHTRCHCGAE |
| NS5B_2609 | DRB1*0401 | LPVAVMGs/rSYGFQYSPGQRV |
| NS5B_2674 | DRB1*1301 | ARVAIk/rSLTERLYVGGPLTN |
| NS5B_2852 | DRB1*0301 | ILMTHFFSv/iLIARDQLEQAL |

Lowercase is putative non-adapted/adapted changes. All peptides based on the genotype 1A associations in Table 2.

**Supplementary Table 4: Sequences used in analyses***

| GT | Protein | N | With >90% coverage of protein |
|---|---|---|---|
| 1A | E2 | 127 | 127 |
| | NS2 | 159 | 143 |
| | NS3 | 154 | 101 |
| | NS4A | 146 | 143 |
| | NS4B | 155 | 91 |
| | NS5A | 147 | 83 |
| | NS5B | 157 | 81 |
| 3A | E2 | 105 | 105 |
| | NS2 | 125 | 106 |
| | NS3 | 113 | 92 |
| | NS4A | 120 | 110 |
| | NS4B | 89 | 63 |
| | NS5A | 110 | 71 |
| | NS5B | 117 | 80 |

**The E2 region targeted in this study did not contain the hypervariable regions.*

**Supplementary Table 5: Clinical and demographic characteristics of co-infected HIV/HCV subjects.**

| ID | Sex | Ethnicity | HLA-A | | HLA-B | | HLA-C | | HLA-DRB1 | | $CD4^+$ cells/ml | Copies HIV/ml |
|---|---|---|---|---|---|---|---|---|---|---|---|---|
| 10004 | M | W | 03:01 | 30:02 | 07:02 | 57:01 | 06:02 | 07:02 | 03:01 | 07:01 | 203 | 50 |
| 10011 | M | AA | 29:01 | 68:02 | 07:02 | 55:01 | 07:01 | 15 | 08:04 | 13:02 | 539 | 7328 |
| 10013 | M | W | 29:01 | 32:01 | 40:02 | 44:03 | 02:02 | 16:01 | 04:08 | 07:01 | 700 | 5118 |
| 10021 | M | AA | | | | | | | 13:01 | 15:03 | 319 | 3576 |
| 10032 | F | W | 26:01 | 30:01 | 13:02 | 57:01 | 06:02 | 06:02 | 03:01 | 07:01 | 543 | 2837 |
| 10039 | F | AA | 23:01 | 30:01 | 42:01 | 44:03 | 04:01 | 17:01 | 03:02 | 07:01 | 416 | 20452 |
| 10042 | M | W | 03:01 | 03:01 | 14:02 | 40:01 | 03:04 | 08:02 | 01:01 | 13:02 | 599 | 50 |
| 10044 | F | AA | 02:01 | 23:01 | 35 | 58:01 | 03:02 | 07:01 | 11:02 | 13:01 | 297 | 49382 |
| 10057 | F | AA | 02:01 | 33:01 | 45:01 | 52:01 | 06:02 | 16:01 | 03:01 | 15:03 | 274 | 11807 |
| 10058 | M | AA | 03:01 | 24:02 | 14:01 | 49:01 | 07:01 | 18:01 | 04:05 | 08:04 | 450 | 50 |
| 10067 | F | AA | 30:02 | 33:01 | 13:02 | 57:03 | 05:01 | 07:01 | 13:02 | 15:03 | 661 | 215 |
| 10077 | F | AA | | | | | | | 03:01 | 11:02 | 210 | 50 |
| 10097 | M | AA | 29:01 | 66:01 | 41:02 | 49:01 | 07:01 | 17:01 | 07:01 | 11:01 | 580 | 36810 |
| 10105 | F | AA | 01:01 | 23:01 | 08:01 | 44:02 | 04:01 | 07:01 | 03:01 | 07:01 | 608 | 8049 |

*M= male, F=female; W = white, AA=African American. Peptides from Supplementary Table 3 were tested on subjects with the relevant HLA type (based on two-digit resolution). Grey indicates subjects with positive IFNγ responses to peptide pools. Note 50 copies HIV/ml was the detection threshold for assay.*

**IFNγ responses in enzyme-linked immunospot (ELISpot) assays to predicted CD4$^+$ T cell epitopes**

PBMC samples were obtained from a subset (n=14) of HCV-exposed individuals recruited from tertiary hospitals in Western Australia between 2006-2012 as previously described [1]. In this study, subjects were selected based on available PBMCs and HLA-DRB1 genotype. These subjects were exposed to HCV via contaminated blood clotting factors (n=12; male subjects with X-linked Haemophilia) or acquired HCV infection via intravenous drug use (n=2) of which two were spontaneous resolvers, three were resolvers following pegylated IFNα/ribavirin treatment and nine subjects were chronically infected with HCV. For these subjects, repeat exposure to diverse HCV strains is common [2,3]. As PBMCs from these subjects were also used to screen for novel CD8$^+$ T cell epitopes based on the same approach [1], we did not have sufficient cell numbers to perform intracellular cytokine staining (ICS) for peptides eliciting a response in the ELISpot assay.

ELISpot assays were performed using the Biomek FX liquid-handling system (Beckman Coulter) as previously described [4] with 200,000 cells/well stimulated with peptides at a final concentration of 10μg/ml. At least one well per assay was allocated as a positive control (anti-CD3 antibody, Mabtech) and three wells as negative (PBMC cells only) controls. A response was deemed positive if the peptide elicited an IFNγ T-cell response at ≥25 spot forming units (SFU)/million PBMCs after background subtraction [5]. The background was defined as the mean plus three times the standard deviation of the number of spots counted in the triplicate negative control wells. For peptides covering predicted epitopes the assay was performed as singletons tested in triplicate.

We tested peptides spanning adapted (non-consensus) and non-adapted (consensus) sequences of nine predicted HLA-DRB1-restricted CD4$^+$ T cell epitopes based on a web-based HLA-peptide binding prediction program (netMHCII; see methods and Supplementary Table 3). Variants of three of these

epitopes have been published before although their HLA restriction was unknown (www.immunoepitope.org). Supplementary Table 6 shows the responses of the subjects to these peptides in ELISpot assays. We were able to confirm four epitopes with positive IFN-γ responses in 7/14 subjects. Importantly, adapted and non-adapted forms of the peptides elicited IFN-γ responses and in some cases the peptide containing the putative adapted site stimulated a stronger response relative to the non-adapted peptide (see Supplementary Table 6 subject 4); similar to our previous report on HIV [6].

For two subjects the predicted $CD4^+$ T cell epitopes tested contained a nested $CD8^+$ T cell epitope for a relevant HLA class I allele within the 15-20mer. However, where $CD8^+$ T cell epitopes in the same region(s) were known, we had previously tested the PBMCs with these known $CD8^+$ T cell epitopes in these subjects in an earlier study and had not detected responses >25 SFU/million PBMCs [1] (Supplementary Table 6).

## Supplementary Table 6: IFNγ ELISpot responses to peptides representing putative HLA-DR-restricted T cell epitopes

| HLA-restriction | Adaptation Protein (position) | Subject | GT/outcome | Peptides (SFU/$10^6$ cells) | | | | Autologous Sequence |
|---|---|---|---|---|---|---|---|---|
| | | | | Non-adapted | Adapted | | | |
| DRB1*11:04^ | NS2 (923) | | | A | V | T | | |
| | | 1 | 1A/Chronic | 80 | 130 | 92 | | T |
| | | 2 | 1A/SVR | 117 | 96 | 53 | | na |
| | | 3 | na/SR | 127 | 137 | 124 | | na |
| DRB1*04:01* | NS5B (2609) | | | S | A | R | | |
| | | 4 | na/SVR | neg | 45 | 160 | | na |
| | | 5 | 1A/Chronic | 120 | 231 | 259 | | S |
| DRB1*13:01*^ | NS5B (2674) | | | K | R | N | T | |
| | | 6 | 1A/Chronic | 169 | 130 | 69 | 115 | I |
| DRB1*03:01* | NS5B (2852) | | | V | I | | | |
| | | 5# | 1A/Chronic | 143 | 146 | | | V |
| | | 6# | 1A/Chronic | 89 | 104 | | | V |
| | | 4 | na/SVR | 36 | 82 | | | na |
| | | 7 | 1B/SVR | 68 | 29 | | | na |

Results are mean from triplicate results. *Similar to known HLA class II-restricted T cell epitope (www.immuneepitope.org). ^No known $CD8^+$ T cell HCV epitope in region for HLA class I alleles carried by subject(s). #Peptides for known HLA class I-restricted T cell HCV epitope(s) tested on subject but response less than 25 SFU/$10^6$ PBMC [1]. Neg = Below threshold of 25 SFU/$10^6$ PBMC. na = Not available. SVR = sustained virological responder (IFN-α/Ribavirin treatment); SR = spontaneous resolver.

1 Pfafferott, K. *et al.* Anti-hepatitis C virus T-cell immunity in the context of multiple exposures to the virus. *PloS one* **10**, e0130420, (2015).

2 Aspinall, E. J. *et al.* Treatment of hepatitis C virus infection among people who are actively injecting drugs: a systematic review and meta-analysis. *Clin Infect Dis* **57 Suppl 2**, S80-89, (2013).

3 Baker, R. I. *et al.* Hepatitis C genotypes in Australian haemophilia patients. *Aust N Z J Med* **26**, 789-792 (1996).

4 Almeida, C. A. *et al.* Automation of the ELISpot assay for high-throughput detection of antigen-specific T-cell responses. *J Immunol Methods* **344**, 1-5, (2009).

5 Lauer, G. M. *et al.* Comprehensive analysis of CD8(+)-T-cell responses against hepatitis C virus reveals multiple unpredicted specificities. *J Virol* **76**, 6104-6113 (2002).

6 Keane, N. M. *et al.* High-avidity, high-IFNgamma-producing CD8 T-cell responses following immune selection during HIV-1 infection. *Immunology and cell biology* **90**, 224-234, (2012).
